# Evidence for the emergence of β-trefoils by ‘Peptide Budding’ from an IgG-like β-sandwich

**DOI:** 10.1371/journal.pcbi.1009833

**Published:** 2022-02-14

**Authors:** Liam M. Longo, Rachel Kolodny, Shawn E. McGlynn

**Affiliations:** 1 Earth-Life Science Institute, Tokyo Institute of Technology, Tokyo, Japan; 2 Blue Marble Space Institute of Science, Seattle, Washington, United States of America; 3 Department of Computer Science, University of Haifa, Haifa, Israel; Stockholm University: Stockholms Universitet, SWEDEN

## Abstract

As sequence and structure comparison algorithms gain sensitivity, the intrinsic interconnectedness of the protein universe has become increasingly apparent. Despite this general trend, β-trefoils have emerged as an uncommon counterexample: They are an isolated protein lineage for which few, if any, sequence or structure associations to other lineages have been identified. If β-trefoils are, in fact, remote islands in sequence-structure space, it implies that the oligomerizing peptide that founded the β-trefoil lineage itself arose *de novo*. To better understand β-trefoil evolution, and to probe the limits of fragment sharing across the protein universe, we identified both ‘β-trefoil bridging themes’ (evolutionarily-related sequence segments) and ‘β-trefoil-like motifs’ (structure motifs with a hallmark feature of the β-trefoil architecture) in multiple, ostensibly unrelated, protein lineages. The success of the present approach stems, in part, from considering β-trefoil sequence segments or structure motifs rather than the β-trefoil architecture as a whole, as has been done previously. The newly uncovered inter-lineage connections presented here suggest a novel hypothesis about the origins of the β-trefoil fold itself–namely, that it is a derived fold formed by ‘budding’ from an Immunoglobulin-like β-sandwich protein. These results demonstrate how the evolution of a folded domain from a peptide need not be a signature of antiquity and underpin an emerging truth: few protein lineages escape nature’s sewing table.

## Author summary

Judged by sequence and structure, about 2,500 different protein evolutionary lineages have been identified to date. Since proteins are central to life, understanding where they came from and how they continue to change is a key challenge in biology. We probed the origins of a relatively recent protein lineage, the β-trefoil, looking at patterns of sequence or structure that are shared between β-trefoils and other protein lineages. On the basis of these shared patterns, we argue that a peptide fragment from the ancient IgG-like β-sandwich evolutionary lineage gave rise to β-trefoils *via* a process we call “peptide budding”. Revealing and understanding cryptic associations between seemingly distinct protein families can shed light on the mechanisms by which new proteins emerge, and contributes to the growing appreciation that the protein universe is highly connected.

## Introduction

Proteins are often approximated as discrete domains from distinct evolutionary lineages. Such classification is powerful, foremost because it can serve as a framework for understanding how proteins within a family change over time, but also because it naturally lends itself to naming and thus aids in communication (as is true for taxonomy in general). Nevertheless, this notion of separability is, ultimately, an approximation: Regardless of whether structure [1,2], sequence [3–6], or both structure and sequence [7,8] are considered, similar segments between proteins that lack global sequence identity are detectable and common. Taken together, these studies emphasize the interconnected, ‘patchwork’ nature of protein evolution [9] and reveal that proteins might be best understood as being comprised of multiple segments, each with its own independent evolutionary history or structural properties. In extreme cases, a protein domain is not unlike the genome that encodes it; that is, a composite of elements that evolves by accretion, exchange, and loss. When these segments are highly conserved within diverse protein families, or overlap with important functional sites, they can provide insight into early protein evolution [8,10] and reveal distant evolutionary relationships [11].

One protein lineage of particular interest as a model system of patchwork evolution is the β-trefoil (ECOD X-group 6), which is characterized by a common ancestor [12] and a pseudo-three-fold axis of rotational symmetry. Although originally proposed to be related to EGF (X-group 389) and ecotin (X-group 521) by gene duplication and fusion [13], proteome-wide sequence analyses [3,6,7] and an experimental fragmentation study [14] have found no support for this hypothesis; instead, the ancestral state of the β-trefoil was most likely a trimerizing peptide that recapitulated the β-trefoil fold. Given that β-trefoils seem to comprise a rare island in an otherwise highly-connected sequence-structure landscape, the origins of the precursor β-trefoil peptide are unclear and potentially the result of a *de novo* emergence event.

Recently, Tenorio and coworkers proposed a link between the β-trefoil and the β-propeller (X-group 5) on the basis of *ab initio* folding simulations [15], in which a four-stranded β-trefoil motif was predicted to adopt a β-propeller blade-like structure by Robetta (the server implementation of the Rosetta protein structure prediction suite) [16]. Although the putative evolutionary relationship between β-propellers and β-trefoils was not validated by a detailed sequence analysis, this study nevertheless provided two valuable insights: First, the elements under consideration were β-trefoil subdomains, not the entire β-trefoil architecture; and second, the subdomains themselves were predicted to be metamorphic (*i*.*e*., adopt different structures in different contexts). Finally, Tenorio and coworkers reignited the >20-year-old debate [13,15] about whether β-trefoils are related to, and perhaps derived from, another common β-protein architecture.

We now report a systematic search for β-trefoil-like sequence segments (bridging themes [11]) and structure motifs (β-trefoil-like motifs) across the known protein universe, foremost to understand the evolutionary history of β-trefoils but also to search for an example of the ‘patchwork model’ of protein evolution. Whereas previous searches have generally considered the β-trefoil architecture in its entirety, we now take a segment-centric approach [17] for improved sensitivity. We observe β-trefoil-like structural motifs in two other protein lineages and identify cases of statistically significant (E-value ≤ 1x10^-3^) sequence segment sharing between a β-trefoil protein and 68 other protein lineages. Taken together, our results demonstrate that the β-trefoil is nowhere near as isolated as previously thought; instead, β-trefoil proteins appear to be a reservoir for sequence innovation in other protein lineages and *vice versa*. The implications of these results with respect to the emergence of the founding β-trefoil are discussed, and we argue that β-trefoils–a relatively recent protein architecture innovation [12,18–20] (**S1 Table**)–are potentially derived from a sequence segment that was once embedded in a different β-protein architecture, specifically, the more ancient [18–20] Immunoglobulin-like β-sandwich (IgG-like β-sandwich; X-group 11). This result is a reminder that lineage emergence from a peptide need not necessarily be taken as a signature of great age but can instead occur by a recent budding event. Ultimately, as our analytical tools become more and more sensitive, it is becoming increasingly clear that, to rephrase John Donne, no protein lineage is an island entire of itself.

## Methods

### Bridging theme search

Bridging themes are sequence segments that link, or bridge, two protein domains that are presumed to be unrelated on the basis of global sequence and structure considerations [6,11]. For the purposes of this report, evolutionary relationships between domains are taken from the state-of-the-art Evolutionary Classification of Domains (ECOD) database [21–23]. Within the ECOD database, X-groups are the highest level of evolutionary-based groupings and referred to here as evolutionary lineages. The criteria for an X-group are intentionally inclusive; thus, while domains within an X-group usually adopt the same architecture, more data may be needed to unambiguously establish a common evolutionary origin. Domains in different X-groups, however, are considered to be entirely distinct evolutionary lineages by ECOD, implying distinct emergence events. Nonetheless, there is ample evidence of bridging themes–cryptic evolutionary associations encoded in short sequence segments–that span different ECOD X-groups [6]. Here, we focus on bridging themes between β-trefoils (X-group 6) and all other protein evolutionary lineages (more than two-thousand X-groups in total).

To build a dataset of hidden Markov models (HMMs) that correspond to structurally relevant parts of the β-trefoil domain, we employed the following procedure: First, we built multiple sequence alignments (MSAs) from the 128 β-trefoil domains in ECOD 70% NR (X-group 6; version develop271) by searching Uniclust30 [24] with HHblits [25] using a coverage cutoff of 90% and a minimum sequence identity of 30% with the master sequence (-cov, -qid flags). For each domain we calculated the residue ranges corresponding to each β-strand with the STRIDE algorithm [26]. Using a sliding window of 4 β-strands (*e*.*g*., β-strands 1–4, 2–5 and so on), which is the length of the repetitive structural element that comprises the β-trefoil architecture, we restricted the MSAs and built HMMs using hmmbuild from the HMMER package [27,28]. Finally, we searched for similarities to all ECOD domains using hmmsearch from the HMMER package. Among the similarities found, we selected the ones with conditional E-values < 1x10^-3^ and not from X-group 6 (the β-trefoils) for further analysis. Sequence similarity searches did not require any degree of structural similarity so that bridging themes between structurally diverse protein architectures could be detected.

To further confirm the statistical significance of the alignment for a given bridging theme, we compared the observed alignment score to an extreme value distribution (EVD) [29] derived from the starting sequence used to generate the HMM profile and the hit identified by HMMER (as in Kolodny and coworkers [6]). The sequences were randomly shuffled and then realigned 1,000 times. The resulting alignment scores were fit to an EVD from which the significance of the score of the observed alignment could be estimated. In the subsequent text and figures, ‘p-value’ refers to this analysis whereas ‘E-values’ are the conditional E-value from the HMMER output. The bridging themes identified in this work are reported in **S1 Data**.

### β-trefoil-like (βTL) motif search

The structural motif used to query the Protein Databank (PDB) was extracted from the sequence-symmetrized β-trefoil called ‘Phifoil’, which is derived from the folding nucleus of human fibroblast growth factor-1 (FGF-1) and adopts an idealized structure [30]. Specifically, residues 52–69 and 77–87 from ECOD domain e4ow4A1 were used for the search motif. The rationale behind this choice of motif is discussed in greater detail in the section **β-Trefoil-like Structure Motifs Occur in Just Two Ancient β-Protein Lineages, Including the IgG-like β-Sandwich**, and relates to the rarity of this motif and its essentiality to β-trefoil structure and folding [31,32]. The search motif was aligned against all domains in the ECOD F99 database (version develop271) with TM-align [33]. TM-scores were normalized to the length of the search motif, which is constant across all alignments. The top hits (excluding β-trefoils) were inspected visually. Alignments to the search motif yielded TM-scores of 0.52 for e2x9wA3 and 0.53 for e2o1cA1. A TM-score > 0.5 is indicative of statistically significant structural similarity [34]. The alignment outputs are included as **S2 Data**.

## Results

### β-trefoils have bridging themes with up to 68 X-Groups, foremost IgG-like β-sandwiches

**Fig 1:**
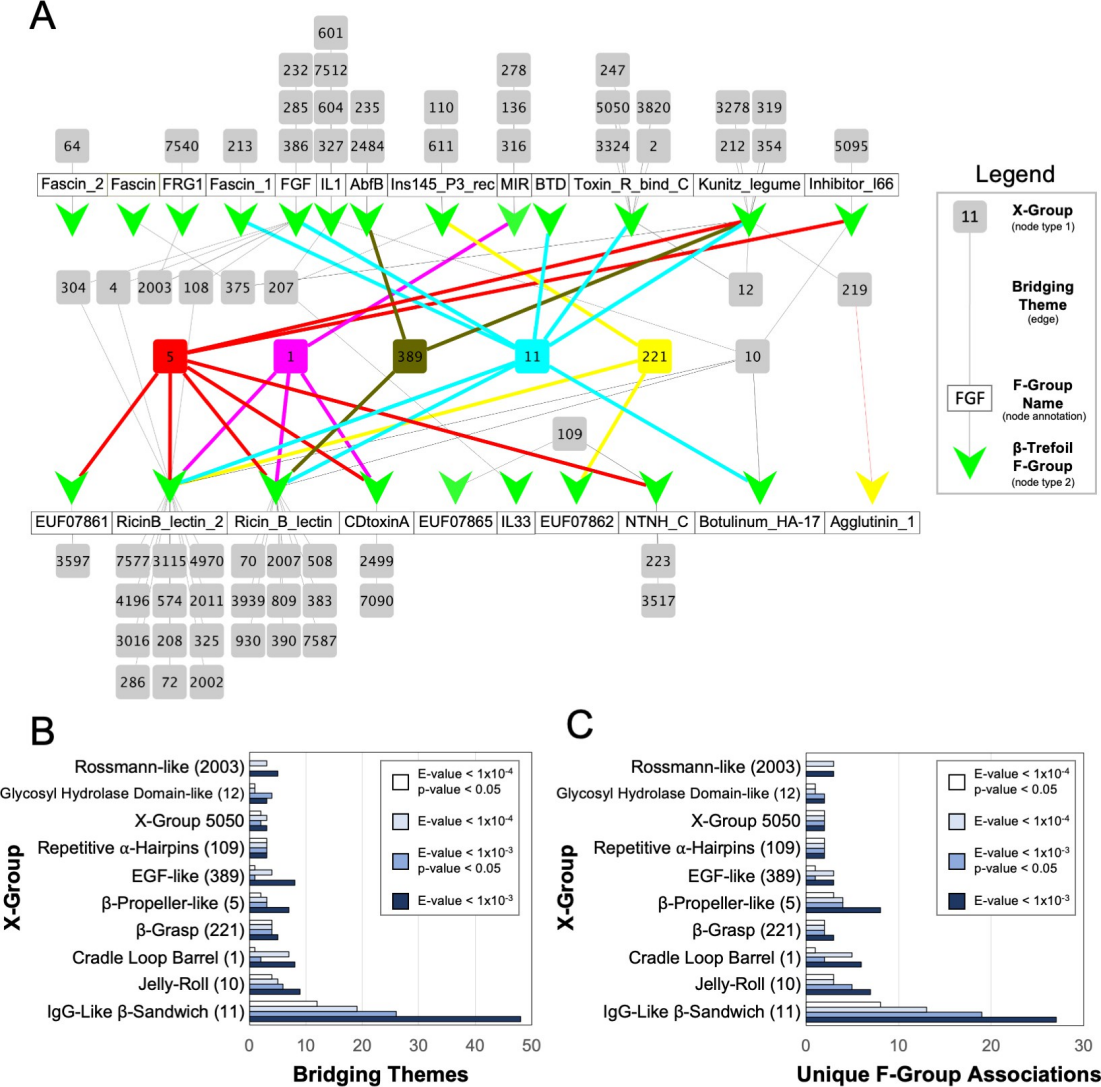
Bridging themes to the β-trefoil evolutionary lineage. **A**. Network of bridging themes (edges) between 23 β-trefoil families (nodes represented by green Vs, defined as an F-group) and 68 other evolutionary lineages (nodes represented by grey and colored boxes, defined as an X-group). The network was constructed using an E-value cutoff of 1x10^-3^. For clarity, evolutionary lineages with a bridging theme to only one β-trefoil family are tiled above or below the name of the associated family (white boxes). Nodes associated with evolutionary lineages of special note are colored as follows: X-group 5 (β-propeller) is red; X-group 1 (cradle loop barrel) in magenta; X-group 389 (EGF) in olive; X-group 11 (IgG-like β-sandwich) in cyan; and X-group 221 (β-grasp) in yellow. The IgG-like β-sandwich is associated with the most β-trefoil families (8 in total). The 7 β-trefoil families for which no bridging themes were found are not drawn. The network graph was rendered with Cytoscape (cytoscape.org) **B**. Bridging theme counts between the β-trefoil and other evolutionary lineages. To account for redundancy, a bridging theme is defined here as an HMM profile that has at least one hit against a non-β-trefoil sequence. IgG-like β-sandwiches are associated with the greatest number of bridging themes to the β-trefoil lineage, even with increasingly stringent statistical cutoffs (see **Methods** for more details). Note that there can be, and often are, multiple, unique bridging themes within a β-trefoil F-group to the same X-group (*i*.*e*., an edge in the network can be associated with more than one shared sequence segment). For clarity, only the top 10 most connected evolutionary lineages are shown. **C**. The number of unique pairwise F-group associations between the β-trefoil and another protein lineage. For clarity, only the top 10 most connected evolutionary lineages are shown.

In total, we found 1083 cases of segment similarity between a β-trefoil and another evolutionary lineage, comprising 68 lineages in total, or about 3% of all evolutionary lineages in ECOD (**Fig 1A**). For cases where all of the bridging theme residues are present in the associated crystal structures (948 cases or 88% of the identified pairs), the RMSD of the structurally aligned segments was calculated (**S1 Fig**). We find that the two structures associated with a given theme are often metamorphic (*i*.*e*., they adopt different conformations) with a median RMSD of 5.6 Å, and only 5.2% of bridging themes having an RMSD < 2.5 Å. The metamorphic quality of bridging themes was observed before in a proteome-wide analysis of sequence sharing events between X-groups [6].

Unsurprisingly, the greatest number of shared themes is with other β-proteins. Among the X-groups that share a theme with the β-trefoil are EGF (first suggested by Mukhopadhyay [13]) and the β-propeller (first suggested by Tenorio and coworkers [15]), with 8 and 7 unique themes, respectively (E-value < 1x10^-3^; **Fig 1B** and **S1 Data**). The evolutionary lineage with by far the greatest number of unique bridging themes to the β-trefoil, however, was the IgG-like β-sandwich, with 48 themes in total. Increasing the stringency of the theme cutoff does not change the result that IgG-like β-sandwiches are the most connected evolutionary lineage to the β-trefoil (**Fig 1B**).

Of the 30 known β-trefoil families (*i*.*e*., ECOD F-groups), 23 have at least one bridging theme to a different evolutionary lineage, with the IgG-like β-sandwich linked to the greatest number of β-trefoil families, 8 in total (E-value > 1x10^-3^; **Fig 1A**). Although β-propellers have a similar number of associated β-trefoil families with 7 (**Fig 1A**), each of these associations is the result of fewer unique sequence themes than with the IgG-like β-sandwich–an average of 1 *vs*. 6, respectively–indicating a weaker evolutionary association. Likewise, the number of unique F-group to F-group associations between the β-trefoil and the IgG-like β-sandwich is greater than with any other X-group (**Fig 1C**).

### The phylogenetic distribution of bridging themes suggests that an early β-trefoil is related to an IgG-like β-sandwich

The distribution of bridging themes across the phylogenetic trees of two protein lineages may help clarify the nature of their evolutionary relationship, though with the caveat that both the rate of sequence sharing and the rate at which sharing events become undetectable (either due to excision of the shared segment or extensive mutation) are unknown. For the purpose of this discussion, we will assume that fragment sharing events are less common than fragment loss events. Consequently, we favor a single early sharing event with some losses over several independent sharing events. On the basis of this assumption, we now provide some possible interpretations for the distribution of shared themes across two protein lineages.

If bridging themes are widely distributed across the phylogenetic trees of both domain lineages (*e*.*g*., all β-trefoils and all Immunoglobulin-like β-sandwiches), it suggests a significant sequence sharing event early in the evolution of both folds, and perhaps a shared evolutionary origin. In cases where the two protein lineages have different ages, if bridging themes are widely distributed across the phylogenetic tree of the younger fold but narrowly distributed across the phylogenetic tree of the older fold, it is consistent with a sequence sharing event that happened very early in the evolutionary history of the younger fold. This early sharing event could (but does not necessarily) correspond to an emergence event. Finally, if the distribution of bridging themes is narrow in both phylogenetic trees, it suggests a more recent theme sharing event, unrelated to the early evolution of either fold. The first and second cases are more consistent with an emergence event than the third case, which would require more loss events.

**Fig 2:**
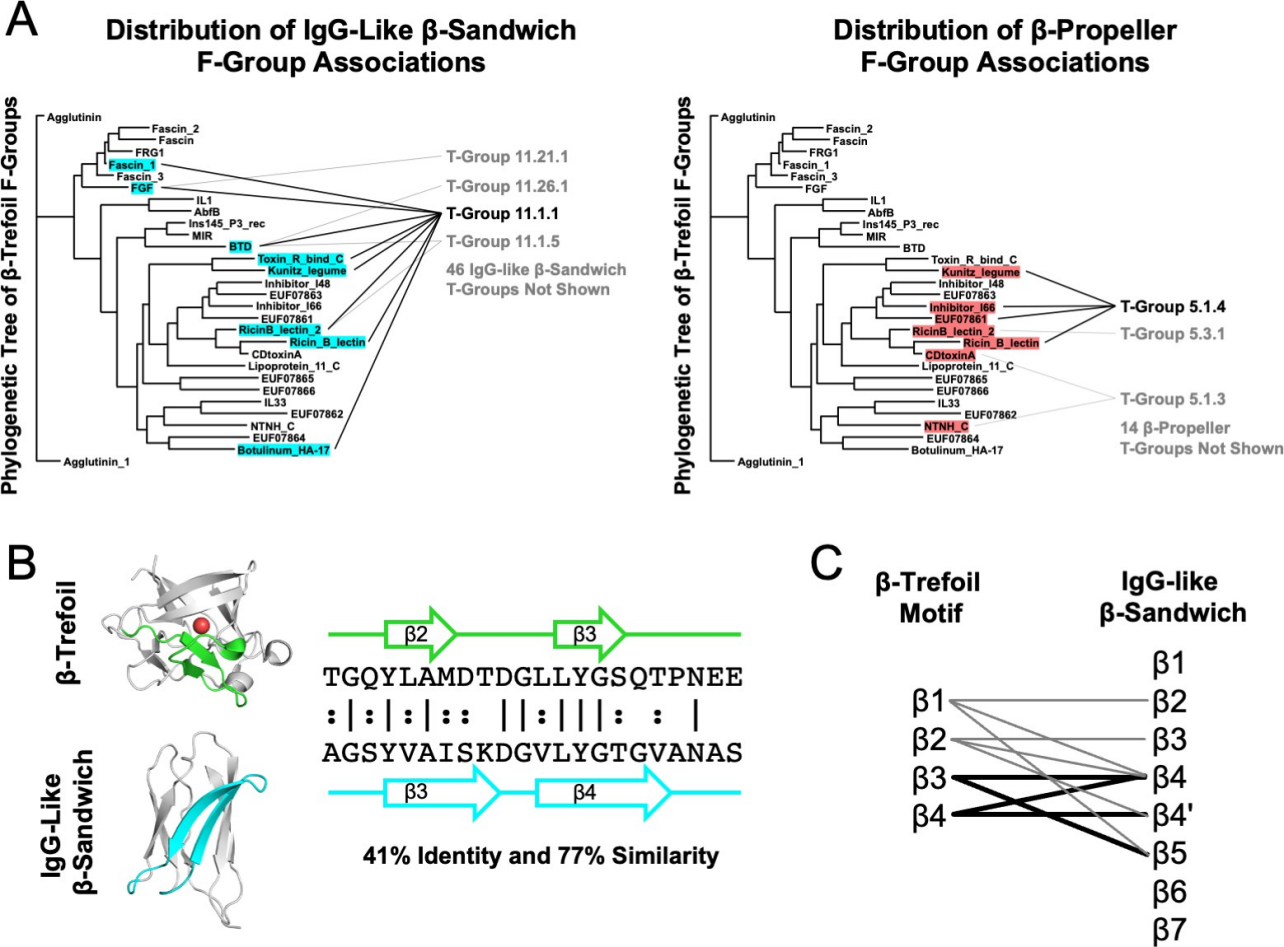
The IgG-like β-sandwich as a candidate progenitor of the β-trefoil. **A**. The distribution of IgG-Like β-sandwich and β-propeller bridging themes across the β-trefoil phylogenetic tree. Β-trefoil families with a bridging theme are highlighted following the color scheme in **Fig 1**. The specific IgG-like β-sandwich or β-propeller T-groups (*i*.*e*., the group of F-groups) that are associated with the β-trefoil family are noted. The IgG-like β-sandwich is not only associated with the most β-trefoil families, these families are well-distributed across the β-trefoil evolutionary tree (more so than the β-propeller, for example). And, while the bridging themes of other protein lineages span multiple T-groups, all of the β-trefoil families associated with the IgG-like β-sandwich also have at least one bridging theme to the same T-group, 11.1.1, which is just one out of 50 T-groups associated with this X-group (though it is the also largest and most diverse one). The β-trefoil phylogenetic tree was adapted from ECOD (http://prodata.swmed.edu/ecod/complete/famtree?tid=6.1.1) with permission. **B**. An example bridging theme between a β-trefoil and an IgG-like β-sandwich, ECOD domains e1rg8A1 and e4rbmA3, respectively. By aligning the themes and denoting the positions of the β-strands, strand associations can be generated. Structure figures were rendered in PyMOL (pymol.org). **C**. Strand associations between the β-trefoil and the IgG-like β-sandwich T-Group 11.1.1 for themes with an E-value < 1x10^-4^ that have a structure model for both sequence segments. Although localized to β2-β5 in the IgG-like β-sandwich structure, the specific strand associations are unclear, perhaps due to rearrangement events in either the β-trefoil and/or IgG-like β-sandwich lineages. Nevertheless, all four β-strands in the β-trefoil repetitive motif have some association to IgG-like β-sandwich. Thin lines correspond to a single bridging theme whereas thick lines correspond to two themes.

The phylogenetic distribution of theme sharing between the more ancient IgG-like β-sandwich and the more recent β-trefoil most closely follows the second case above (**Fig 2A**, left panel): A phylogenetically wide distribution of β-trefoil F-groups are associated with just a single T-group (that is, a group of F-groups; specifically, 11.1.1) of IgG-like β-sandwiches, an evolutionary lineage with 50 T-groups in total. Note that **Fig 2A** renders the phylogenetic tree of β-trefoil families (F-groups) provided by the ECOD database, whereas the other evolutionary lineage (*i*.*e*., IgG-like β-sandwiches or β-propellers) is represented as a list of T-groups. Contrast this phylogenetic distribution with that of the β-propeller bridging themes (**Fig 2A**, right panel), the evolutionary lineage with the second most associations to β-trefoil F-groups. In this case, the most prominent T-group (5.1.4) is associated with just four β-trefoil F-groups, and these F-groups are more closely related to each other than those associated with the IgG-like β-sandwich, consistent with a more recent sharing event. For another example, see the distribution of β-grasp bridging themes (**S2 Fig**). Indeed, the IgG-like β-sandwich is unique in the breadth of associations across the β-trefoil phylogenetic tree that is achieved by just a single T-group. However, it should be noted that within the IgG-like β-sandwich evolutionary lineage, X-group 11.1.1 is the largest and most sequence diverse. The fact that detectable signals are not present in all β-trefoil families may relate to the complex evolution of this lineage [35] or to the fact that β-trefoils generally have highly diverged sequences [12].

In cases where the sequences associated with the bridging theme were present in the structure models of both domains, strand associations between the IgG-like β-propeller and the β-trefoil were identified to determine if they are consistent across the bridging themes (**Fig 2B** and **2C**). Although the strand associations implied by the theme alignments are not internally consistent–that is, there is no simple one-to-one correspondence between strands in the IgG-like sandwich and strands in the β-trefoil–they are localized to a specific region of the β-sandwich structure (β2-β5) and, between them, encompass all four β-strands of the β-trefoil (the structure of which is described in greater detail the next section). Although none of the identified themes adopt a β-trefoil-like structure within an IgG-like β-sandwich domain, perhaps such a structure has arisen *de novo* or is no longer detectable at the sequence level. If a β-trefoil-like motif can be identified within the IgG-like β-sandwich, it would demonstrate the potential of this evolutionary lineage to serve as a structural scaffold.

### β-trefoil-like structure motifs occur in just two ancient β-protein lineages, including the IgG-like β-sandwich

**Fig 3:**
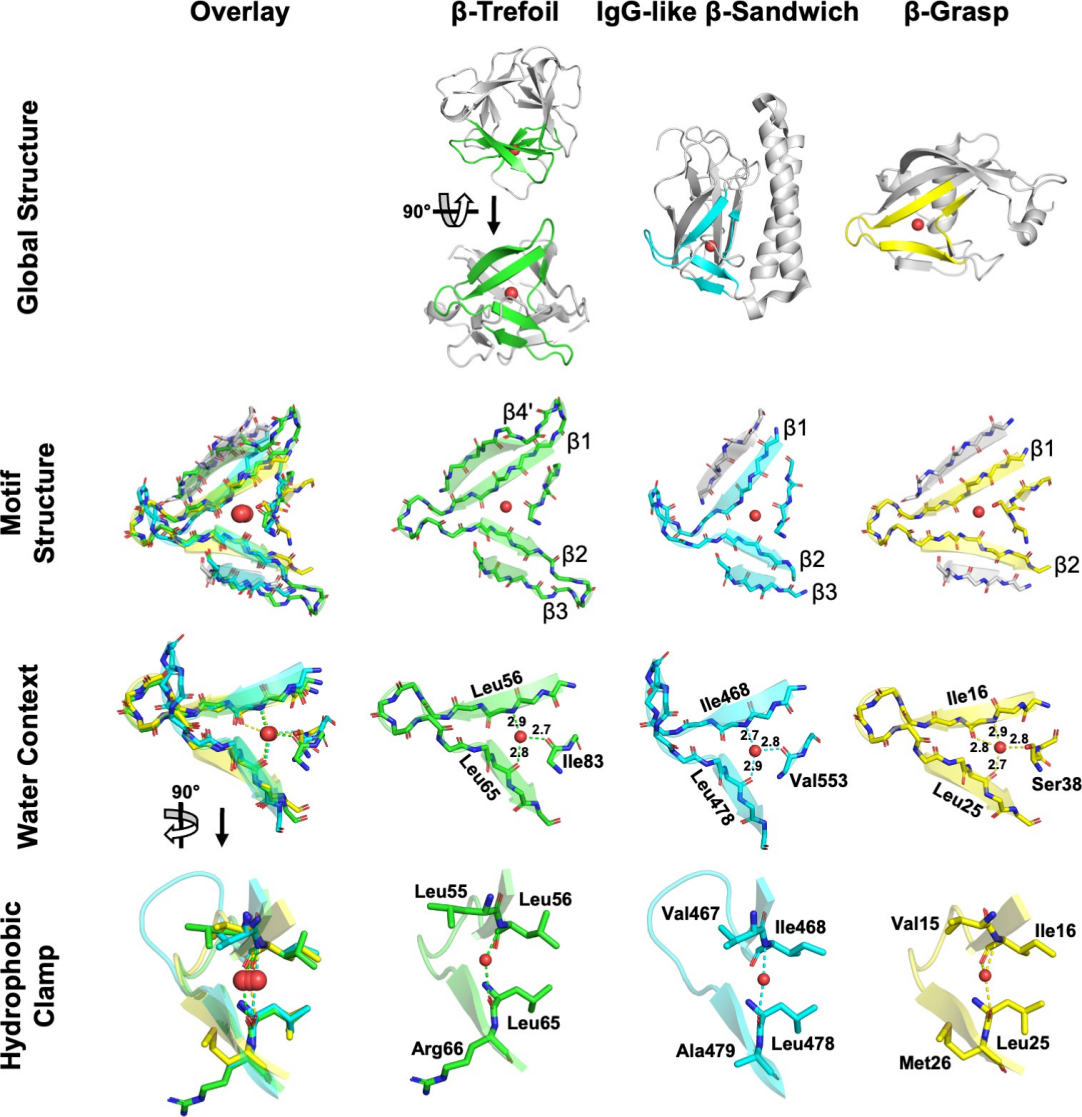
β-trefoil-like (βTL) motifs across the protein universe. The canonical β-trefoil motif (second column, green) comprises four β-strands, their intervening loops and turns, and a conserved water molecule. Shown here is a circular permutation of the canonical motif definition, comprised of β4’, β1, β2, and β3 and the subsequent loop, where β4’ refers to the final β-strand (β4) of the previous β-trefoil motif. Water molecules are indicated by a red sphere. β-strands shaded grey in the **Motif Structure** panel are surrogate β-strands, donated by other parts of the host domain, and are not part of the βTL motif proper but satisfy the same role as a canonical β-strand. Distances in the **Water Context** row are shown in Angstroms (Å). In the **Global Structure** panel, C-terminus of the β-grasp domain has been truncated for clarity. The structure motifs shown here were extracted from the following ECOD domains: β-trefoil: e3o4bA1 (F-group 6.1.1.8); IgG-like β-sandwich: e2x9wA3 (F-group 11.1.4.227); β-grasp: e2o1cA1 (F-group 221.4.1.3).

We next sought to investigate whether structures similar to β-trefoil motifs exist elsewhere in the protein universe. The β-trefoil architecture is comprised of three symmetrically juxtaposed structural subdomains referred to as β-trefoil motifs. Each β-trefoil motif is made up of four β-strands and an absolutely conserved buried water molecule (**Fig 3**; first identified in [31] and confirmed during the course of this study). The conserved water molecule forms hydrogen bonds to three distinct backbone sites, two β-strands and a loop, and is a hallmark structural feature that fundamentally enables the β-trefoil motif and architecture. Recently, the conserved water has been shown to make significant contributions to both the structure of the folding nucleus and the overall enthalpy of folding [32]. To search for β-trefoil-like motifs in other protein lineages, we focused on the conserved water molecule and the structural elements that interact with it, namely β-strands β1, β2, and the loop after β3 (canonical β-trefoil motif strand numbering). We define this structural motif as the minimal definition of the β-trefoil-like motif (βTL) because, unlike a simple β-hairpin [2], we expect this feature to be rare, if not unique to the β-trefoil fold altogether. To reduce family-specific bias, the structure used for searching was extracted from an idealized β-trefoil protein with fully symmetrized sequence and structure [30] (see the **Methods** for more details).

Structural alignments against the representative domains for each lineage of the ECOD database identified two independent protein lineages bearing a βTL motif (**Fig 3**). In both cases, the similarity of the identified βTL motif to a *bona fide* β-trefoil motif extends beyond the search motif itself to include β3 or β4’ (the last β-strand, β4, of the preceding β-trefoil motif, which packs against β1; see **Fig 3 Motif Structure**). Unlike the β-trefoil architecture, which is rotationally symmetric, the detected βTL motifs were found in two asymmetric architectures–the β-grasp (ECOD X-group 221) and the IgG-like β-sandwich. Despite divergent structural contexts, all of the βTL motifs adopt a highly similar conformation (**Fig 3 Motif Structure**) and conserved water position (**Fig 3 Water Context**). In each case, the residues on β1 and β2 that form a backbone hydrogen bond with the conserved water also form a ‘hydrophobic clamp’ with their sidechains (**Fig 3 Hydrophobic Clamp**). Both evolutionary lineages associated with a β-trefoil-like motif also share bridging themes with β-trefoils. In the case of the IgG-β-sandwich, these bridging themes do not overlap with the βTL motif identified here, though there is some notable sequence similarity to an extant β-trefoil domain. For the β-grasp, however, there is partial overlap between a bridging theme and a β-trefoil-like motif. The novel βTL motifs are now described in turn.

#### IgG-like β-sandwich

The occurrence of a βTL motif in the IgG-like β-sandwich evolutionary lineage was identified in ECOD F-group 11.1.4.227 –a different T-group than that associated with the majority of the bridging themes (11.1.1). The fact that structure comparisons and sequence comparisons identified different protein elements is consistent with the observation that the shared themes we identified are often metamorphic (*i*.*e*., adopt different conformations in in different contexts). Similar βTL motifs were not detected in closely related F-groups, or any other F-group within the IgG-like β-sandwich evolutionary lineage. As in the canonical β-trefoil motif, the IgG-like β-sandwich βTL motif is characterized by a conserved water bound by three short (<3.0 Å) hydrogen bonds to the protein backbone. The IgG-like β-sandwich βTL motif comprises β1, β2, β3 and the water-binding loop of the canonical β-trefoil motif. Although β4’ is missing, a β-strand from elsewhere in the protein packs against β1, effectively acting as surrogate β4’. β2 and β3 are positioned to form a tight hairpin, as in the canonical motif, if not for the presence of an α-helix inserted into the connecting turn. Although this domain was not identified in our bridging theme analysis, a sequence comparison between the search motif and this βTL motif reveals a five-residue stretch, GQYLA, that is located at an equivalent position in both structures (on β3 and the subsequent loop).

#### β-grasp

Nudix-related hydrolases are a collection of F-groups within the β-grasp evolutionary lineage that comprise T-group 221.4.1. Both a bridging theme (**S2 Fig**) and a βTL structural motif (**Fig 3**) were identified in this T-group. Although the structures associated with the bridging theme are different, they do overlap at what is roughly β1 of the canonical β-trefoil motif (**S3 Fig**). The core βTL motif present in β-grasp proteins comprises only β1, β2, and the water-binding loop–and is thus shorter than that of the IgG-like β-sandwich–strands exist on either side of the motif occupying the role of β3 and β4’ of the canonical motif. βTL motifs within the Nudix-related hydrolases are characterized by a conserved water molecule between β1 and β2. However, unlike β-trefoils and IgG-like β-sandwiches, the water-binding loop of Nudix-related hydrolases can interact with the conserved water *via* the hydroxyl of a serine sidechain. This new interaction allows the water molecule to reposition and form four short hydrogen bonds to the protein (**Fig 3**). In another structural twist, the βTL motif of NDX2 from *Thermus thermophilus* HB8 [36] lacks the conserved water molecule altogether. This ‘dry’ βTL motif achieved by the presence of proline residues, the backbone of which cannot act as a hydrogen bond donor (**S4 Fig**). No such dry βTL motif could be identified in natural β-trefoils or IgG-like β-sandwiches.

## Discussion

### An updated protocol reveals bridges to an island architecture

We, and others [4,8], have previously analyzed patterns of global sequence fragment sharing across the protein universe, both with [7] and without [6] structural constraints. In all cases, connections to the β-trefoil were either marginal or absent. Why, in the present analysis, do we uncover extensive evidence of fragment sharing between β-trefoils and other protein lineages? We attribute the improved sensitivity of our approach to three methodological changes: First, our analysis more directly considers β-trefoil fragments (*i*.*e*., a sliding window of 4 β-strand elements, which is the length of the repetitive motif that forms the β-trefoil architecture; see **Methods** for more details) and not the β-trefoil structure as a whole. When the entire structure–that is, three consecutive β-trefoil motifs–was used, searches were strongly biased in favor of other β-trefoils, thus giving the impression that this evolutionary lineage is an island. This approach was motivated both by the accepted models of β-trefoil evolution, in which the β-trefoil emerged from a single oligomerizing β-trefoil motif [12,14], and previous studies that attempted to uncover associations between β-trefoils and other lineages [15]. Second, we generated HMM profiles from a 70% identity cluster of modern β-trefoils. Consequently, we emphasized somewhat more recent evolutionary events than others do (*e*.*g*., Alva and coworkers [8]) in our models of the β-trefoil families. Finally, by using HMMER for this analysis, we could compare β-trefoil HMM profiles to the sequences of all ECOD domains (over 730,000 sequences in total). As such, uncommon or under sampled sequence features in the non-β-trefoil evolutionary lineages, and signatures of parallel evolution between lineages [11] could be detected. Ultimately, HMMER did identify meaningful alignments to both the β-trefoils themselves (which is trivial) and to segments in other lineages.

For repeat proteins like the β-trefoil, and for protein lineages that lack a highly conserved functional feature, these methodological improvements appear to be crucial to understanding early evolutionary processes. We now know that β-trefoils are active participants in sequence fragment sharing across the protein universe, a fundamental shift from the perspective that this fold originated *de novo* and is isolated in sequence space.

### The origin of β-trefoils

Experimental fragmentation studies have demonstrated that the β-trefoil architecture can be realized by a trimerizing 42-residue peptide, begging the question: Where did the founding peptide come from in the first place? Is it possible that the β-trefoil is a derived from another protein lineage, perhaps akin to the evolutionary relationship between flavodoxins and TIM barrels [37]? Given that β-trefoils are relatively young (**S1 Table**), they emerged in a cell already populated by numerous complex proteins, which may be a prerequisite for this evolutionary model. But which evolutionary lineage was most likely to be the parent lineage?

**Fig 4:**
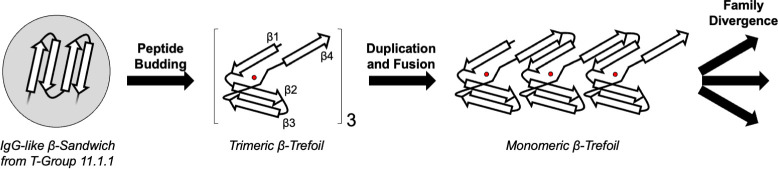
Emergence and evolution of the β-trefoil architecture. The peptide budding model hypothesizes that the first β-trefoil emerged when an excised segment from an IgG-like β-sandwich (grey shaded circle) trimerized to form a β-trefoil. This model of emergence is suggested by a preponderance of bridging themes between IgG-like β-sandwiches and β-trefoils (**Fig 1**), their wide phylogenetic distribution among β-trefoils but narrow distribution among IgG-like β-sandwiches (**Fig 2A**), and the observation of a βTL motifs in a modern IgG-like β-sandwich protein (**Fig 3**). The homo-trimeric β-trefoil was then duplicated and fused into a single domain, events predicted by Ponting and Russell [12] and demonstrated experimentally by Lee and Blaber [14]. The founding monomeric β-trefoil then gave rise to multiple β-trefoil families by both whole domain duplication and *via* a budding-like process of β-trefoil motifs, as described by Broom and coworkers [35]. As drawn, the embedded fragment in the IgG-like β-sandwich does not adopt a β-trefoil motif-like structure and is comprised of four β-strands. However, we do not exclude the possibility that the embedded peptide adopted a β-trefoil-like motif or that it was shorter than a canonical β-trefoil motif and underwent a duplication concurrent with the budding event.

Previously studies have suggested potential evolutionary associations with EGF proteins or β-propellers, both of which are observed here. However, on the basis of the analysis presented above, the most likely fragment donor for a founding β-trefoil peptide was the IgG-like β-sandwich, an evolutionary lineage that i) predates the β-trefoil [18–20] (**S1 Table**); ii) has the strongest signatures of theme sharing with the β-trefoil, with ~6 times more shared themes (10^−3^ E-value cutoff) than any other evolutionary lineage (**Fig 1**); iii) exhibits shared themes with a wide phylogenetic distribution on the β-trefoil side and a comparatively narrow phyletic distribution on the IgG-like β-sandwich side (**Fig 2A**); and iv) possess a rare example of structural motif convergence, a feature detected in only 0.1% of X-groups (**Fig 3**). Taken together, these observations present the strongest evidence to date for a derived β-trefoil evolutionary model (**Fig 4**), in which (1) the nascent β-trefoil peptide emerged in the context of an IgG-like β-sandwich protein, and then (2) ‘budded’ from the parent protein to form a standalone, independently folding trimeric β-trefoil that was ultimately (3) consolidated onto a single chain by gene duplication and fusion events. Evolution from a fragment, therefore, may be property of a very ancient protein lineage [8] (*cf*. Ferredoxins [38], Rossmanns [11], P-Loops [39], and the (HhH)_2_-fold [40]) but may also be a property of a more recent protein lineage, especially ones with a repetitive or symmetric architecture.

Berezovsky and colleagues noted a preponderance of short closed-loop structural elements, in which the protein backbone loops back onto itself (defined as segment end-points less than 10 Å apart), and showed that these fundamental structural motifs are often associated with function [41]. β-proteins, therefore, can be understood as chains of sequential closed-loop structural elements, namely, β-hairpins [2]. Although β-hairpin structure alone is too simple to imply homology (given the limited space of possible structures for short segments) the intuition derived from the analysis of Berezovsky and colleagues is consistent with our proposed evolutionary model: β-hairpins from one β-protein giving rise to the β-hairpins of another β-protein. The extent to which similar processes may have occurred early protein evolution, but are no longer detectable in sequence, is unknown.

Finally, we note that the budding of a peptide from a folded domain to found a new protein family has been observed before: Broom and coworkers [35] argued that a single β-trefoil motif from a monomeric β-trefoil protein can be duplicated and fused to yield a new family of β-trefoils, a process of ‘modular evolution’. Likewise, the helix-hairpin-helix motif is known to exist as both an embedded functional element and as a standalone domain with a two-fold axis of rotational symmetry, demonstrating that transitions between embedded structural elements and independently folding symmetric domains occurs elsewhere in the protein universe [8,42].

## Conclusions

Modern proteins are a web of evolutionary associations that manifest across various levels of organization, from genes to domains to sequence segments. Advancements in search algorithms and analysis protocols, such as the bridging themes presented here, will undoubtably uncover even more associations between globally unrelated evolutionary lineages–revealing that seemingly isolated folds, like the β-trefoil, are in fact active participants in fragment sharing. Characterizing the presence and extent of bridging themes between evolutionary lineages can provide valuable insight into protein domain emergence [8,11]; in the present case, by suggesting that the β-trefoil is a derived fold that arose from an IgG-like β-sandwich.

## Supporting information

S1 Data Bridging themes to the β-trefoil evolution lineage identified in this study. See the **Methods** section “Bridging Theme Search” for additional details.
(XLSX)

S2 Data TM-align output from which the β-trefoil-like motifs were identified. See the **Methods** section “β-Trefoil-like (βTL) Motif Search” for additional details.
(XLSX)

S1 Fig Metamorphic character of bridging themes. Distribution of RMSD values of bridging themes for cases where both sequences associated with the bridging theme are present in their respective crystal structures.
(PDF)

S2 Fig The distribution of β-grasp bridging themes across the β-trefoil phylogenetic tree. (PDF)

S3 Fig An overlapping β-trefoil-like motif and bridging theme. Regions of the domains associated with the bridging themes are colored green or yellow. The conserved water molecule of the βTL motif is shown as a red sphere. ECOD domains are e2vseA2 (β-trefoil F-group RicinB_lectin_2) and e2azwA1 (β-grasp T-group 221.4.1). The C-terminus of the β-grasp domain has been truncated or clarity.
(PDF)

S4 Fig β-trefoil-like motif without a conserved water in the β-grasp evolutionary lineage. **A**. In the canonical β-trefoil motif, a conserved water bridges β1 and β2 (red sphere), a feature that is retained in many Nudix hydrolases. Shown here is ECOD domain e2o1cB1 (F-group 221.4.1.3). **B**. In some Nudix hydrolases, however, proline residues preclude water binding. In ECOD domain e2yvpA2 (also F-group 221.4.1.3), formation of a ‘dry’ β-trefoil-like motif results in two unsatisfied backbone carbonyls (annotated with red circles).
(PDF)

S1 Table Fold ages of some relevant X-groups taken from reference [19]. (DOCX)
