# Supplementary figures and images for: Evidence for the emergence of β-trefoils by ‘Peptide Budding’ from an IgG-like β-sandwich
Source: PLoS Comput Biol. 2022 Feb 14;18(2):e1009833. doi: 10.1371/journal.pcbi.1009833 (PMC8880906; doi:10.1371/journal.pcbi.1009833)

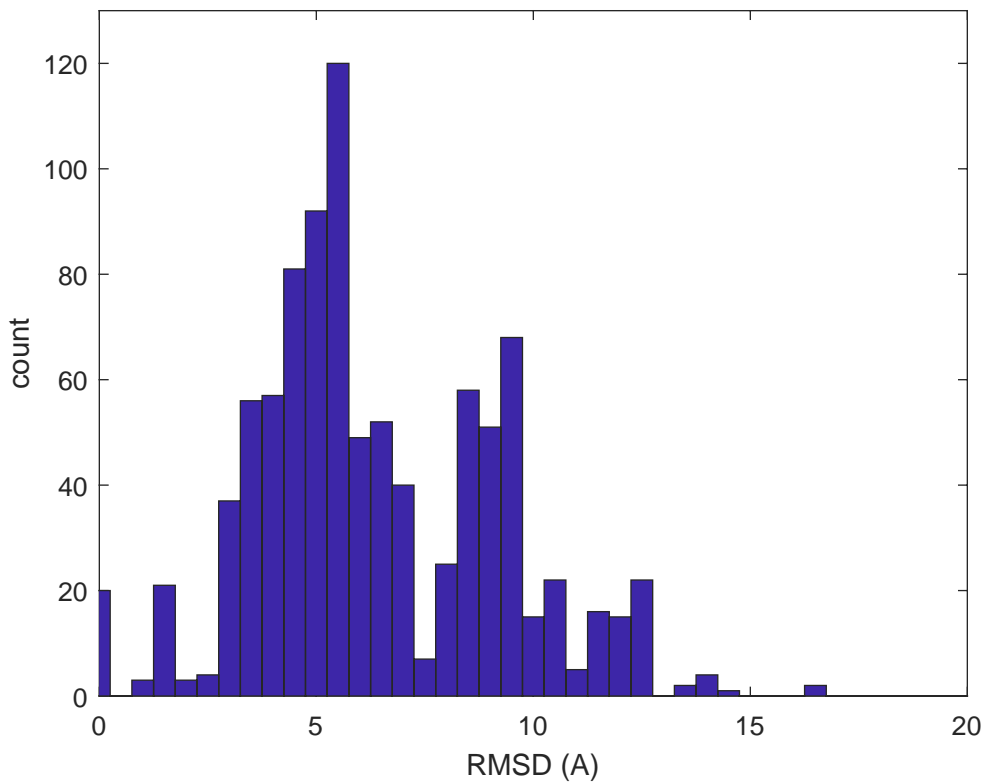

Supplement: S1 Fig — Distribution of RMSD values of bridging themes for cases where both sequences associated with the bridging theme are present in their respective crystal structures. (PDF) [file pcbi.1009833.s003.pdf]

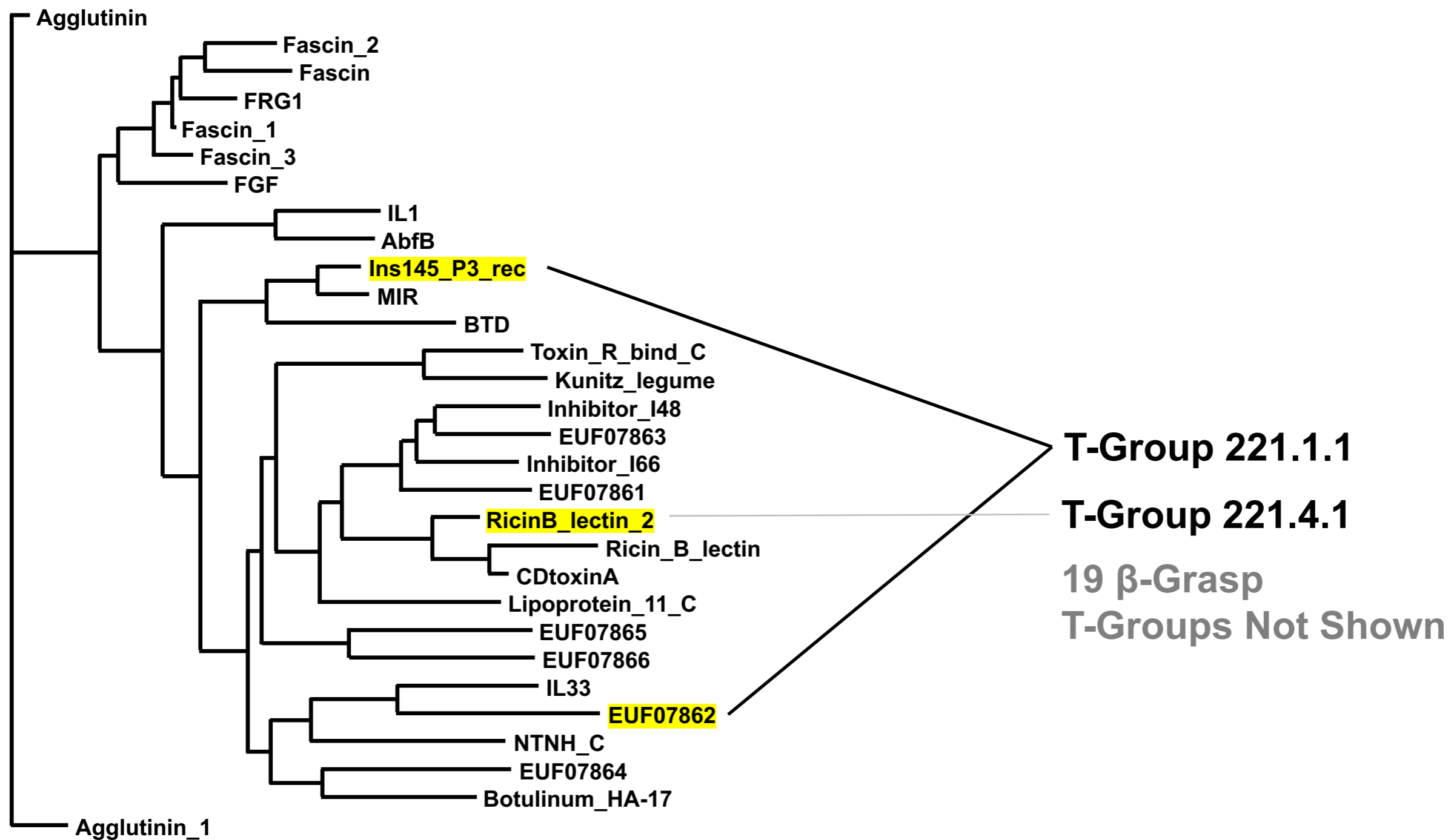

Supplement: S2 Fig — (PDF) [file pcbi.1009833.s004.pdf]

$\beta$ -Trefoil

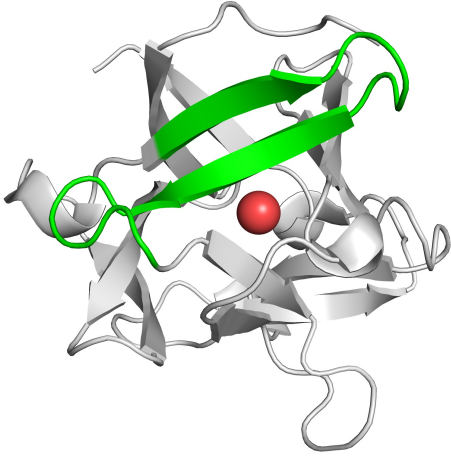

$\beta$ -Grasp

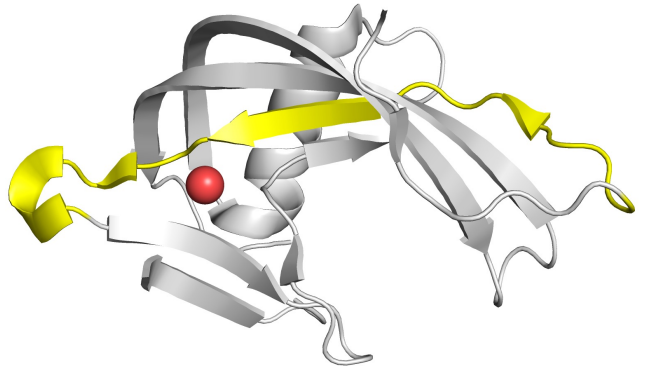

Supplement: S3 Fig — Regions of the domains associated with the bridging themes are colored green or yellow. The conserved water molecule of the βTL motif is shown as a red sphere. ECOD domains are e2vseA2 (β-trefoil F-group RicinB_lectin_2) and e2azwA1 (β-grasp T-group 221.4.1). The C-terminus of the β-grasp domain has been truncated or clarity. (PDF) [file pcbi.1009833.s005.pdf]

A

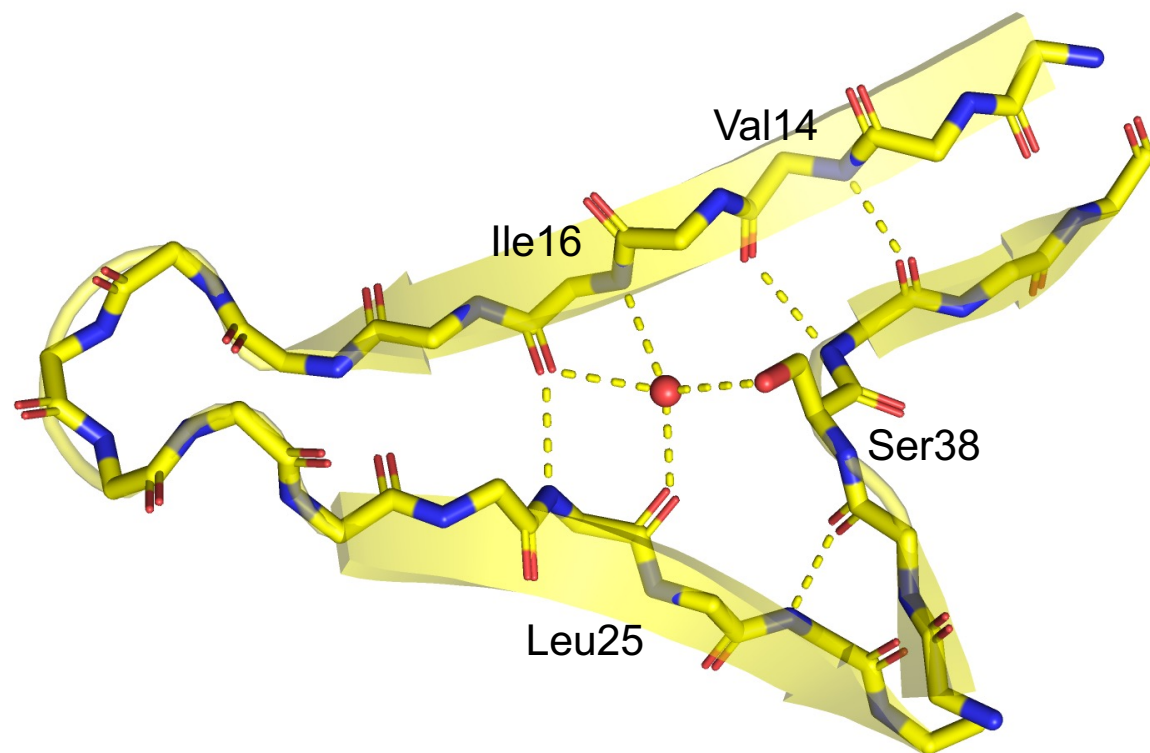

**“Wet”  $\beta$ -Trefoil-like Motif**

B

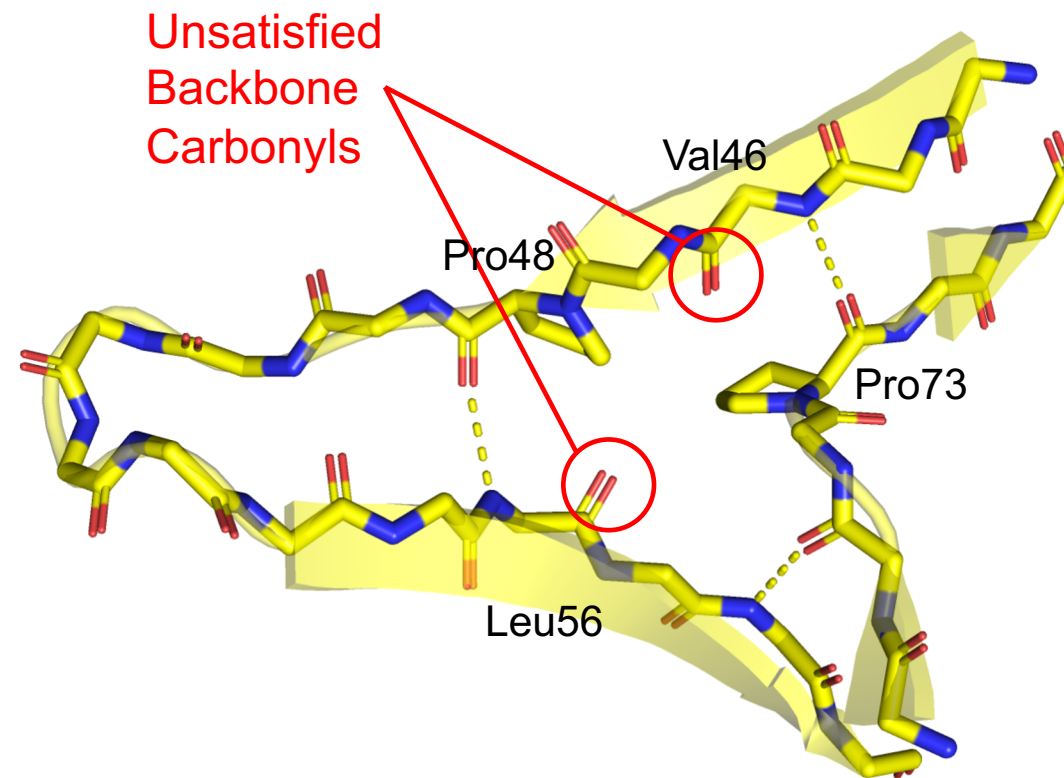

**“Dry”  $\beta$ -Trefoil-like Motif**

Supplement: S4 Fig — A. In the canonical β-trefoil motif, a conserved water bridges β1 and β2 (red sphere), a feature that is retained in many Nudix hydrolases. Shown here is ECOD domain e2o1cB1 (F-group 221.4.1.3). B. In some Nudix hydrolases, however, proline residues preclude water binding. In ECOD domain e2yvpA2 (also F-group 221.4.1.3), formation of a ‘dry’ β-trefoil-like motif results in two unsatisfied backbone carbonyls (annotated with red circles). (PDF) [file pcbi.1009833.s006.pdf]
