# Supplementary material for: Evidence for the emergence of β-trefoils by ‘Peptide Budding’ from an IgG-like β-sandwich
Source: PLoS Comput Biol. 2022 Feb 14;18(2):e1009833. doi: 10.1371/journal.pcbi.1009833 (PMC8880906; doi:10.1371/journal.pcbi.1009833)
Supplement: S1 Table — (DOCX) [file pcbi.1009833.s007.docx]

**Table S1.**

| **X-Group** | **SCOPe Fold** | **SCOPe Fold Name** | **Fold Age (maximum parsimony) [1]** | **Fold Age (fusion parsimony) [1]** |
| --- | --- | --- | --- | --- |
| 6 | b.42 | beta-Trefoil | 0.6-0.79 | 0.6-0.79 |
| 11 | b.1 | Immunoglobulin-like beta-sandwich | 1 | 1 |
| 10 | b.29 | Concanavalin A-like lectins/glucanases | 1 | 1 |
| 1 | b.49 | Domain of alpha and beta subunits of F1 ATP synthase-like | 1 | 1 |
| 221 | d.15 | beta-Grasp (ubiquitin-like) | 1 | 1 |
| 5 | b.69 | 7-bladed beta-propeller | 1 | 1 |
| 389 | g.3 | Knottins (small inhibitors, toxins, lectins) | 0.6-0.76 | 0.6-0.76 |
| 109 | a.118 | alpha-alpha superhelix | 1 | 1 |
| 5050 | f.38 | MFS general substrate transporter | 1 | 1 |
| 12 | b.30 | Supersandwich | 1 | 1 |
| 2003 | c.2 | NAD(P)-binding Rossmann-fold domains | 1 | 1 |

**References**

1. Edwards H, Abeln S, Deane CM. Exploring Fold Space Preferences of New-born and Ancient Protein Superfamilies. PLoS Computational Biology. 2013. doi:10.1371/journal.pcbi.1003325
